# Aphorisms on the Analysis of the Human Mind

**Published:** 1818-02

**Authors:** Thomas Parkinson


*For the London Medical and Physical Journal.*

### *Aphorisms on the Analysis of the Human Mind;* by THOMAS PARKINSON, M.D.

BEFORE I enter upon my purpose of giving some aphorisms on the Analysis of the Human Mind, I beg leave to observe, that, in the Table, I have kept all the GENERA of solids distinct from one another, showing that the GENERA of solids were associations of one CLASS of solids with another CLASS, as—

**Myeleuthytona Hæmeuthytodes.** Association of muscle with nerve, as the retina, &c.

**Enyphanta Hæmeuthytodes.** Association of muscle with membrane, as the intestines, &c.

**Or, associations of the fourth CLASS of solids with other substances, which the CLASS itself secretes, as,—**

**Enyphanta Collodes.** Association of membrane with albumen, which albumen the membrane itself secretes; and the same with bone, adipose substance, &c.

**Or, particular forms and configurations, as—**

**Stereoses.** Solid.

**Siphonodes.** Tubulated.

**Siphogyrodes.** Tubulated and convoluted, &c.

It appears, that there are only four abstract solids (CLASSES) in the whole animal composition. These I have distinguished and denoted by such terms as refer to the characteristic peculiarities of each CLASS; I here mean the anatomical peculiarities, or those marks by which we distinguish one CLASS of solids from another. In forming and denoting the GENERA, I have been careful to employ such adjectives as relate and point to the nature of the GENUS, as,—

**Hæmeuthytona Stereoses; Hæmeuthytona Antrodes.** Dependent upon form or shape.

**Enyphanta Coniodes.** Dependent upon an association with a peculiar substance secreted by a membrane.

**Myeleuthytona Hæmeuthytodes.** Dependent upon an association of one abstract solid with another, &c.

Now, I must observe, that, in order to be logical in the Analysis of the Human Mind, so as to afford an intelligible view of its construction and composition, it is not only requisite to understand the two first CLASSES of Solids with their GENERA, together with the *physiology* of each GENUS, but also to appoint appropriate and determinate terms to denote the individual elements of which the mind is composed, and carefully limit each term to its proper situation and use.

The Elements of the Human Mind are,—

1. PERCEPTION.
2. CONCEPTION.
3. IDEA.
4. JUDGMENT.
5. MEMORY.
6. VOLITION.

PERCEPTION is the simple or first feeling of any thing by an Organ of Sense, and consists of Sensation and Motion, the two essential attributes of animal life; arising out of two distinct kinds of organization of animal matter, viz.—Sensation is the function of Myeleuthytona. Motion is the function of Hæmeuthytona. And they are required to be associated, to afford the faculty of perceiving; not only in the immediate organs of sense, but in the brain within the head.

CONCEPTION is the first process in the Phreneteliosis, and consists of the association of a number of Perceptions.

IDEA is the second process in the Phreneteliosis, and consists of the association and arrangement of a number of Conceptions.

JUDGMENT is the third process in the Phreneteliosis, and consists of the association, arrangement, and connexion, of a number of Ideas.

Judgment is the Maximum of the Mind; but Mind has other faculties or properties, viz.—

MEMORY, which is the fourth process in the Phreneteliosis, consists of the Retrogression of Judgments into Ideas; of Ideas into Conceptions; and of Conceptions into Perceptions.

VOLITION, which is the fifth process of the Phreneteliosis, and consists of the transmission of the commands of the mind to Hæmeuthytona, or the moving solid, through Myeleuthytona Enyphantodes, or the nervous cord: it may, perhaps, be better understood by the delivery of the Mind's will to the Muscles, by the movements of which the precise will of the Mind is known, and judgments are distinguished.

I may, perhaps, be allowed a short digression, to show the close and beautiful analogy this Analysis of the Mind bears to that of Uterine Functions.

1. PERCEPTIONS. The excitants of the uterus.
2. CONCEPTIONS. Impregnated uterus.
3. IDEAS. Utero-gestation.
4. JUDGMENTS. Commencement of parturition.
5. MEMORIES. Processes of parturition.
6. VOLITIONS. Fœtal emancipation.

To return,—this appears to me the most rational method of analysing the human mind that our present knowledge of anatomy and physiology affords, or in which it can serve as our guide.

In contemplating the mind, as well as any other anatomical or physiological subject, it is necessary to assign certain limits to the *capabilities* of *abstract parts*, and to give to each its relative value in the composition of the whole, that we may avoid confusion and error.

But, when Dr. Hartley advances a proposition, "that the white medullary substance of the brain,—the spinal marrow, and the nerves proceeding from them,—is the immediate instrument of sensation and motion;" and such a proposition is supported by the high authority of Dr. Priestley and others; whilst it is conceded to by physicians and anatomists; and is pretended to be confirmed by appeals to nosological facts,—it is not to be wondered that the proposition of Dr. Hartley should be suffered to be at rest.

It behoves us, however, in the present improved state of medical science, to enquire whether the proposition itself be correct, and if the hypotheses founded upon it are not obnoxious to well-grounded objections.

Has the white medullary substance of the brain, the spinal marrow, and the nerves proceeding from them, as an aggregate, the power of performing Motion? It appears to me to possess the faculty of Sense only,—all we know of Sense is Perceptions carried to the brain, there elaborated into Conceptions, Ideas, Judgments, Memory, and Volition; the two latter of which are reactions of the mind.

The maximum of the mind is JUDGMENT; MEMORY is the Retrospect of Judgment; VOLITION is the Transmission of Commands from the Judgment to the Moving Agents, Hæmeuthytona, or Muscle.

The express and only function of Hæmeuthytona is Kinesis,—direct action; it does not possess the capability of reaction; its only Physiology is Direct Action and Rest; hence the necessity and almost universality of antagonist muscles.

Hæmeuthytona, or the Muscle, will,—it must,—be in a state of rest until it is commanded into Action by Myeleuthytona, or rather by the Judgment or Mind, through the medium of Myeleuthytona Enyphantodes, or the Nervous Cord.

I must repeat, that Myeleuthytona is limited to the function I have ascribed to it,—that of Æthesis and Æthesphoresis—that is to Perceptions—Elaborations into Mind—Transmissions to and from the Brain, viz. of Perceptions *to* the Brain—of commands of the mind to the moving solids, *from* the Brain.

It is evident, then, that a nerve can no more do the office of a muscle, than a muscle can do that of a nerve. It requires something more than nerve to perform motion; it requires Hæmeuthytona, or the muscular solid, whose immediate and only function is Kinesis, or Direct Action.

Dr. Hartley, and his commentators, should have been informed, that voluntary motion will just as certainly be impaired, or entirely lost, by the interruption or abolition of the function of a muscle, as it will be by the interruption or abolition of the function of a nerve.

The plain truth is, the faculties of these two abstract solids are as different as their construction and composition.

Myeleuthytona, the nerve, gives commandment to Hæmeuthytona, the muscle,—and Hæmeuthytona, or muscle, obeys it, so long as it has power so to do. But yet, although the commands are regularly carried to the moving solid, they cannot be obeyed any longer than the capability of the moving solid is preserved, notwithstanding the commands are transmitted, and the desire of the mind is that those commands should be faithfully obeyed.

It is only by assigning to abstract parts their respective and natural functions, and properly associating them, that we can obtain any just notions of the laws of the animal economy.

How absurd then must be Dr. Hartley's proposition, "the white medullary substance of the brain, the spinal marrow, and the nerves proceeding from them, is the immediate instrument of Motion?"

Such a proposition being adopted and advocated by high authorities, and acceded to by eminent physicians and anatomists, naturally leads to erroneous reasoning in support of false theories. Truth, under such circumstances, must remain in obscurity; and, the only way in which we can approach her, is that of resisting all propositions and hypotheses founded upon them, which cannot be demonstrably proved to rest upon solid facts.

To conclude, I shall not impose upon the understanding any hypothetical propositions, to account for the phenomena of animal life, by interposing some invisible, unintellible something, as the *primum mobile* of the nerves. Electricity, Galvanism, Magnetism, &c. have been suggested as the probable intermediates between organized animal matter and vitality. Has Electricity, or any other subtile fluid, Intelligence or Volition?—In what consists the excellence of a subtile fluid, to entitle it to nearer relation to divinity than the most highly exalted modification of animal solids?

I shall cease to make any further enquiry on this subject; but shall repeat, that Perception, Conception, Idea, Judgment, Memory, and Volition, are the component parts or elements of the Human Mind; and that the mind is formed by a regular progression of Perceptions, Conceptions, and Ideas, into Judgment.—This is MIND.

The faculties of the Mind are Memory and Volition.—Memory is the regression of Judgment into Ideas, of Ideas into Conceptions, of Conceptions into Perceptions.

Volition is the power the Mind has of sending her commands, through the nerves to the moving agents.

I do not pretend to explain the *modus agendi:* that is, and, perhaps, for ever will be, concealed from us.

I rest upon those facts which are demonstrable and evident, and humbly submit, under the misery of the Human Mind, to Him who is the author of it.

*Leicester; Dec.* 15, 1817.

